# Making older women more visible in healthy ageing

**DOI:** 10.1016/j.lanwpc.2026.101891

**Published:** 2026-05-29

**Authors:** The Lancet Regional Health – Western Pacific

The Western Pacific region has the largest and fastest-growing population aged 60 years or older in the world, with women accounting for more than half. Despite the large number of older women living in the region, their health needs and care experiences remain insufficiently discussed.

Designing and tailoring policies and interventions for older adults should be based on appropriate data and evidence. In this issue, Chenkai Wu and colleagues systematically summarise community-based longitudinal ageing studies in east and southeast Asia. Although there is geographical imbalance in data availability, with most data centred in Japan, Mainland China, Singapore, and South Korea, the majority of included cohorts collected participants' sociodemographic information, used objective measures to check older adults' physical performance, and about half collected biospecimens for laboratory analysis. Progress in community-based longitudinal ageing cohort studies in east and southeast Asia reflects methodological and technical advancement, benefiting from the region's continued investment and collaborative work such as the Action Plan for Successful Ageing in Singapore and Health China 2030.

These efforts should be further expanded to address gender inequality in healthy ageing. Women have longer life expectancy but spend a larger proportion of their later years living with illness, poverty, and social isolation than men. These disadvantages on later-life health and wellbeing reflect the cumulative inequality and discrimination women experience throughout their lives in education, employment, health care, and social protection. In the Asia–Pacific region, older women are often the main informal care providers, yet they are less likely to be covered by pension systems. They may also have greater needs for health care and long-term care partly due to longer life expectancy, as well as higher risk of diseases such as cardiovascular disease, stroke, depression, and dementia associated with menopause and postmenopausal changes. Changes in hormones alongside major life transitions can also affect mental health, which is closely linked to physical health and the care they need. Moreover, symptom manifestations often differ even for the same disease between men and women.

Despite differences in health needs and social support, older women remain largely under-represented in health interventions and policy making. This systemic exclusion results from inequalities in women's power, knowledge, and access to information. The continuing increase in the number of older women in the Western Pacific region pushes us to rethink how to plan and deliver health and social care for this underserved population. Reliable data infrastructure systematically documenting gender-disaggregated, gender-sensitive, and gender-specific information is the first step to reflect women's physical, mental, and socioeconomic changes across ageing.

It is therefore necessary to develop and validate sex-specific and gender-specific, as well as culturally tailored, indicators that might not be captured by instruments primarily developed in European populations. Accordingly, data collection should adopt a life-course perspective and integrate data from sources beyond those focused solely on ageing populations. Many Asian adults who have recently reached older adulthood experienced war, famine, and displacement earlier in life, which can fundamentally shape their health and wellbeing in later years. Historical contexts should be considered to better understand the long-term effects of early-life traumas and potential intergenerational effects on offspring. To ensure that collected data are meaningful to older women, they should be engaged during study design and data collection to capture both the vulnerabilities and strengths for ageing. Qualitative data should also be incorporated to deepen understanding of quantitative findings and may also be useful to address sensitive but quite often neglected topics such as domestic violence and sexual health. For scientific studies, it would be beneficial to integrate sex and gender information throughout the research process by following and contextualising the Sex and Gender Equity in Research (SAGER) guidelines.

The International Day of Action for Women's Health on May 28 calls for efforts to highlight emerging political, social, and economic factors affecting women's health and to monitor progress. Gender-disaggregated data are essential to formulating and implementing gender-responsive strategies that provide a more comprehensive and inclusive understanding of population ageing. In addition to coordinating regional initiatives to establish a common set of core measures for key constructs and to improve data-sharing and funding mechanisms, we should apply a gender lens on data collection to support healthy ageing for both men and women across the Western Pacific region.

